# Impact of Resistance on Therapeutic Design: A Moran Model of Cancer Growth

**DOI:** 10.1007/s11538-024-01272-6

**Published:** 2024-03-19

**Authors:** Mason S. Lacy, Adrianne L. Jenner

**Affiliations:** School of Mathematical Sciences, Queensland University of Technology, Brisbane, QLD Australia

**Keywords:** Moran model, Resistance, Cancer, Treatment

## Abstract

Resistance of cancers to treatments, such as chemotherapy, largely arise due to cell mutations. These mutations allow cells to resist apoptosis and inevitably lead to recurrence and often progression to more aggressive cancer forms. Sustained-low dose therapies are being considered as an alternative over maximum tolerated dose treatments, whereby a smaller drug dosage is given over a longer period of time. However, understanding the impact that the presence of treatment-resistant clones may have on these new treatment modalities is crucial to validating them as a therapeutic avenue. In this study, a Moran process is used to capture stochastic mutations arising in cancer cells, inferring treatment resistance. The model is used to predict the probability of cancer recurrence given varying treatment modalities. The simulations predict that sustained-low dose therapies would be virtually ineffective for a cancer with a non-negligible probability of developing a sub-clone with resistance tendencies. Furthermore, calibrating the model to in vivo measurements for breast cancer treatment with Herceptin, the model suggests that standard treatment regimens are ineffective in this mouse model. Using a simple Moran model, it is possible to explore the likelihood of treatment success given a non-negligible probability of treatment resistant mutations and suggest more robust therapeutic schedules.

**Supplementary Information:**

The online version contains supplementary material available at 10.1007/s11538-024-01272-6.

## Introduction

Resistance of cancer cells to chemotherapy is the first cause of cancer associated death and overcoming resistance is a critical focus for many researchers (Cui et al. 2020; Nedeljković and Damjanović 2019; Pokhriyal et al. 2019; Desbats et al. 2020). Resistance to cancer therapeutics is multifaceted and can arise due to several factors (Vasan et al. 2019). Some cancers are intrinsically resistant to treatments due to genetic complexity (Wang et al. 2019) and other cancer types can develop resistance during treatment (Schmitt et al. 2016; Karantanos et al. 2013). Drugs can be selected to target a specific cancer type, which presents a lower probability of developing a resistance to that drug (Nikolaou et al. 2018). Overall, more research into the impact of treatment resistance is desperately needed to improve our ability to overcome or better treat cancer patients.

**Fig. 1:**
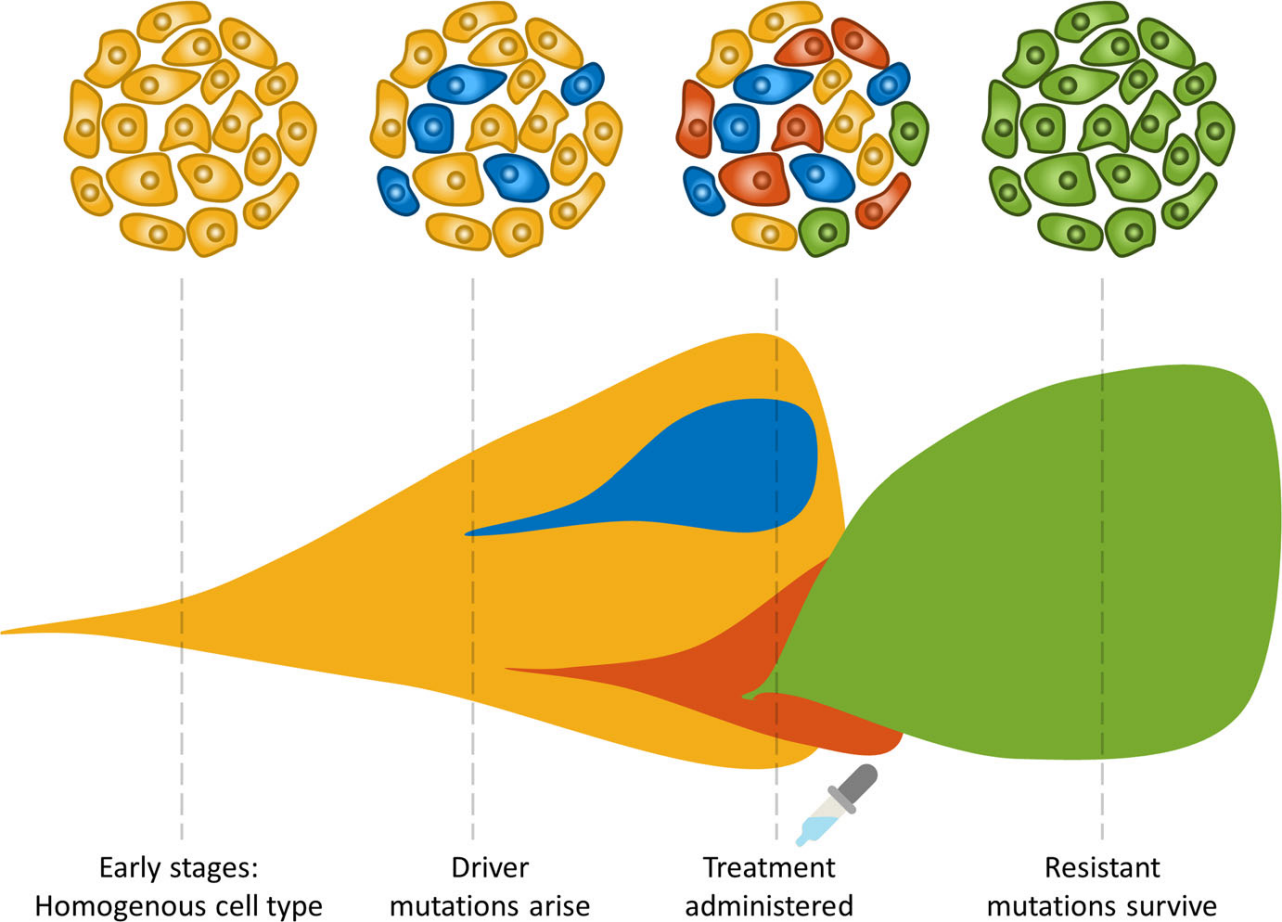
Schematic depicting tumour evolution over time and the fixation of a resistant clone following therapy. As a cancer grows, it is subjected to various pressures which can cause mutations to arise. Some clones may contain mutations that may be more adept at coping with treatment or provide a fitness advantage to those cells, such as faster proliferation rates, and we denote these as driver mutations. After treatment, often cells with driver mutations conferring resistance and/or fitness advantages will expand in number. In some cases, this can result in a tumour that is no longer as genetically complex. Most importantly, these tumours are often no-longer sensitive to the original therapy (Color figure online)

Resistance to targeted therapies can arise from selective growth of pre-existing subclones within the bulk of the tumour that carry drug-resistance mutations, and thus have a survival advantage (Schmitt et al. 2016) (Fig. 1). For example, some breast cancer cells will overexpress the HER2 protein on the cell surface. This overexpression is due to a driver mutation that accumulates over time (Ng et al. 2015; Marin 2023). A drug known as Herceptin is able to target cells overexpressing HER2 and induce death (Lewis Phillips, et al. 2008). Unfortunately, a significant number of patients do not benefit from this therapy, highlighting the need to understand the mechanisms of cancer resistance (Pohlmann et al. 2009) and the impact of therapeutic protocol design.

Standard clinical protocols for cancer treatment, such as chemotherapy, typically employ the maximal drug dose that can be tolerated by the patient, often referred to as the Maximum Tolerated Dose (MTD) (Kareva et al. 2015). The administration of chemotherapy at reduced doses given at regular, frequent time intervals, termed ‘metronomic’ chemotherapy, presents an alternative to standard MTD (Kareva et al. 2015). In a similar manner, adaptive therapy is an approach to treatment based on maintaining a proportion of a treatment-sensitive population and including treatment holidays, where no drug is administered (Gatenby et al. 2009; Lin-Rahardja et al. 2023). Often, a single systemic administration (or infusion intravenously) can result in significant systemic toxicity and only a fraction of the injected dose reaching the tumour. As such, there has been a growing interest in the development of localised targeted delivery systems which can modify the biodistribution of drugs (Rezk et al. 2019; Nazir et al. 2021; Kearney and Mooney 2013). These include biodegradable implants (Padmakumar et al. 2018) or nanocarriers (Natarajan et al. 2014). Often these sustained-release systems provide a lower drug dosage over a longer period, however, not much is known about their success in the presence of resistant mutations (Chen et al. 2019; Brioli et al. 2014). More research into minimising the effects of drug resistance is required, highlighting the need to implement and test novel methods in simulated environments.

Unsurprisingly, cancer growth is an extremely heterogeneous disease, both in the way it presents across the human population, and in the inherent cellular subclones of a tumour (Fig. 1). Cancers are usually comprised of multiple cell types, often referred to as clones, that can differ by the passenger and driver mutations they contain. Clones can give rise to specific cell lineages and the variation within a tumour is referred to as intra-clonal heterogeneity (Yates and Campbell 2012). This presence of clones in a cancer cell population results in behaviour similar to that of Darwin’s evolution theory, which can promote drug resistance as clones compete for dominance (Brioli et al. 2014). Treatment can also facilitate this behaviour by causing the death of some cancer clones which could be benign, and supporting the more rapid growth of resistant and aggressive clones (McGranahan and Swanton 2017), so care must be taken during therapy.

Mathematical modelling has been used for many years to capture cancer growth and treatment resistance (Craig et al. 2020; Altrock et al. 2015). While it is possible to use deterministic models to capture this physical system (Yin et al. 2019; Jackson and Byrne 2000), stochastic mathematical modelling provides a way to more accurately account for the inherent randomness present in cancer growth and treatment (Wilkinson 2009; Ditlevsen and Samson 2013). Some of the stochastic techniques used to model cancer resistance include branching processes (Zhang et al. 2023; Clapp and Levy 2015), birth–death processes (Iosifescu et al. 2013; Allen 2010), Wright-Fisher models and agent-based models (Klowss et al. 2022; Metzcar et al. 2019; Wang et al. 2015). A Moran process is a stochastic model that considers a fixed population of size $N$ with a fixed number of states within the population. Transitions between states in each time step is governed by some probability. Moran models are often used for modelling cancer growth, which is driven predominantly by cell division and death (West et al. 2016a, b; Heyde et al. 2021), and are able to capture the interactions between healthy and cancerous cell clones using different fitness advantages $f$ (Altrock et al. 2015).

In evolution, cells or animals are considered to hold a fitness advantage if some inherent characteristics provide them with a benefit. For example, cancer cells may be considered more fit if they are a product of a driver mutation that provides them with an ability to proliferate more rapidly. In Moran models, cell fitness affects the probability of a particular cell being selected to reproduce, which could lead to fixation of a more fit cell type. West et al*.* (2016a) present a Moran model that considers multiple clones of both healthy and cancerous cells. Here, passenger mutations provide transitions between different healthy cell states, where some of these healthy cells have a higher probability of providing a driver mutation to cancerous cells. They also consider applying concepts seen in the prisoner’s dilemma evolutionary game to select the fitness for healthy and cancerous cells. They identify that treatment should be administered in the early stages of the cancers growth to prevent genetic complexity. Similarly, Heyde et al. (2021) finds that the Moran process accurately describes the driver mutations found in hematopoietic stem cells.

Moran models are also used to track the probability of tunneling, which occurs when a cancer mutant reaches fixation before the original cancer. Komarova (2006), Haeno et al. (2013) and Jackson et al. (2014) each considered a Moran model with three cell types, where the third type is a mutation from the original cancer with higher fitness. Each of these models discover that tunneling occurs naturally at some rate depending on the fitnesses of the cancerous and mutated cells. Similar Moran models have been applied to create more complex stochastic models that consider spatial information about the cells, like ‘warlock’ (Bak et al. 2023), or the one-dimensional model presented by Komarova (2006). This one-dimensional spatial Moran process models the generation of cancer mutants more accurately than the non-spatial model, which seems to underestimate these mutations. Werner et al*.* (2011) used a two type Moran model to capture wild type leukemic cancer cells sensitive to Imatinib and Imatinib resistant cancer cells. They compared the dynamics of their model to in vitro experiments to determine the fitness of different cell populations. Altrock and Traulsen (2004) determine analytical expressions for the fixation probabilities of a two cell Moran model.

Moran modelling to date has been insightful for standard dosing regimens. In this work, we investigate the effects of resistance in MTD versus sustained low-dose treatment. To do this, we develop a simple Moran model to capture the development of chemotherapy resistance during cancer growth and treatment. In Sect. 2, we detail the modelling assumptions and experimental data used in this work. In Sect. 3, we investigate the effects of different treatment modalities and discuss the implications of our findings for future cancer treatments.

## Methods

### Moran Birth–Death Process

**Fig. 2:**
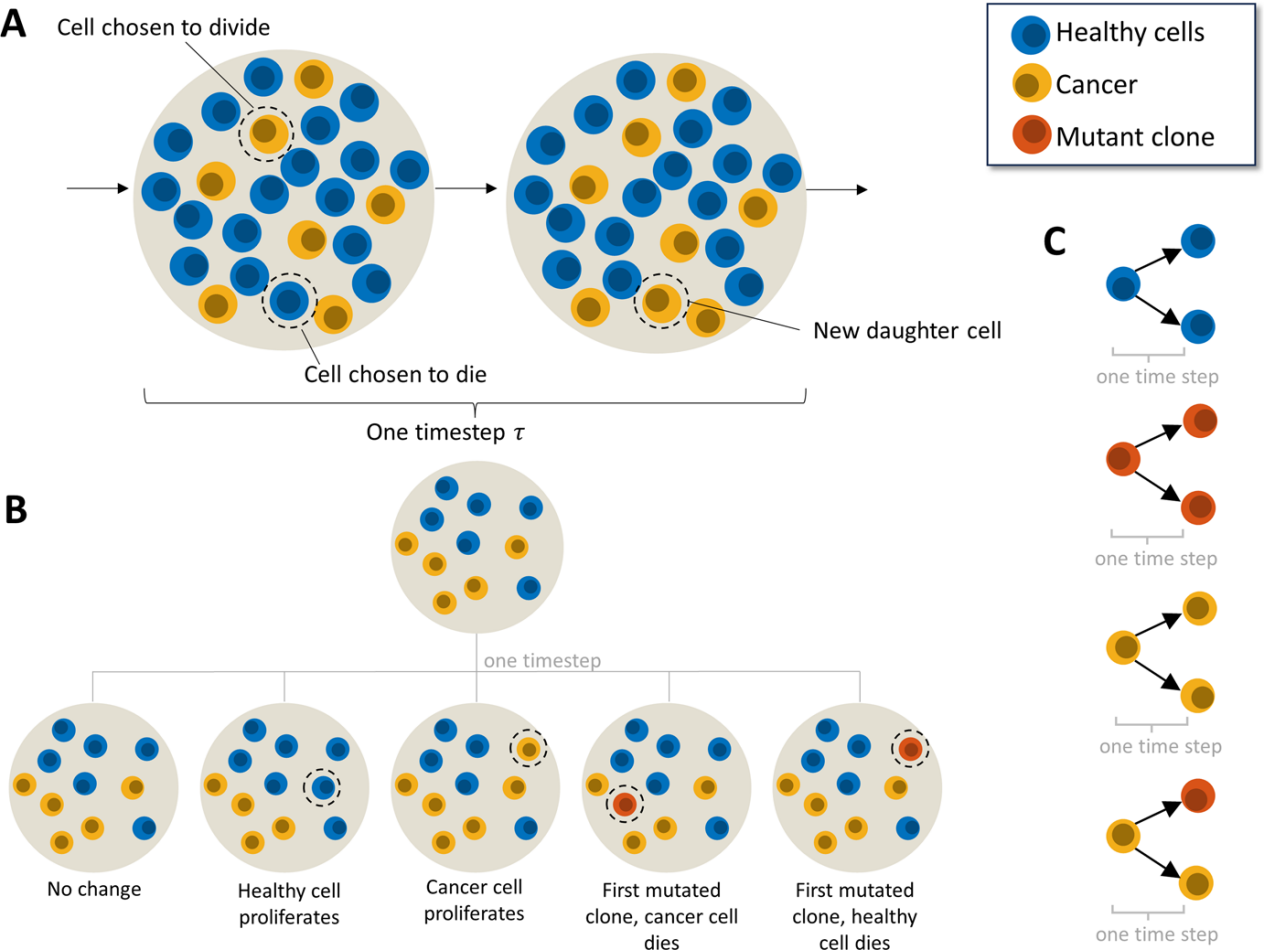
Moran model for cancer cell growth and mutation. **A** We assume initially there are two populations of cells: healthy and cancerous. In a single timestep $\tau$ one cell is chosen to divide and one cell is chosen to die based on the probabilities given in Eq. (1). The cell chosen to die is replaced with a cell of the type chosen to proliferate. **B** Including mutations into the model (red cell) initially gives rise to 5 possible outcomes in a single time step where only healthy and cancerous (non-mutated) reside in the population. Either there is no change in the population, due to a cell of the same type being chosen to divide and die, a cancer cell is chosen to proliferate, and a healthy cell is chosen to die so the cancer population grows, a mutated clone is created and replaces a healthy cell, or a mutated clone is created and replaces one of the original cancer cells. **C** Lineage rules for each individual cell type. Healthy cells can divide into healthy cells, cancer cells can divide into cancer cells or mutant cells, and mutant cells can divide into mutant cells. Note that, for division to occur, a cell of a different type must be selected to die (Color figure online)

The Moran process is a Markov process that models stochastic dynamics of a population with constant size $N$. In this work, we present a Moran model that describes the interactions between healthy cells and different clones of cancerous cells (Fig. 2 and Figure S1). Considering in the initial stages of cancer growth there are two cell types, cancerous (type $c$) and healthy (type $h$), with corresponding finesses ${f}_{c}$ and ${f}_{h}$. The populations of each cell type (${N}_{c}$ and ${N}_{h}$ respectively) can be tracked in any given simulation, where the total population of cells remains constant at $N={N}_{c}+{N}_{h}$. In a single timestep of this two-cell Moran model, there are three possible outcomes (1) cell type $c$ proliferates and cell type $h$ dies, (2) cell type $h$ proliferates and cell type $c$ dies, or (3) cell type $c$ and $h$ undergo no change. Each option has an associated probability:

1 $$ \begin{aligned} & \left( 1 \right) \quad P\left( {N_{c} \to N_{c} + 1,N_{h} \to N_{h} - 1} \right) = \frac{{N_{c} f_{c} }}{{N_{c} f_{c} + N_{h} f_{h} }} \cdot \frac{{N_{h} }}{N} , \\ & \left( 2 \right)\quad P\left( {N_{c} \to N_{c} - 1,N_{h} \to N_{h} + 1} \right) = \frac{{N_{h} f_{h} }}{{N_{c} f_{c} + N_{h} f_{h} }} \cdot \frac{{N_{c} }}{N}, \\ & \left( 3 \right)\quad P\left( {N_{c} \to N_{c} ,N_{h} \to N_{h} } \right) = 1 - \frac{{N_{c} N_{h} \left( {f_{c} + f_{h} } \right)}}{{N\left( {N_{c} f_{c} + N_{h} f_{h} } \right)}}, \\ \end{aligned} $$

where the probability of each transition is proportional to the probability of a cell of a particular type being chosen to reproduce and a cell of a particular type being chosen to die. Specifically, we assume the probability of being selected to die is proportional to the relative size of the cell’s type in the total population $N$. In contrast, the probability of a cell being chosen to reproduce is proportional to its fitness $f$. Formulating the probabilities in this manner is similar to previous work by West et al*.* (2016a), Altrock et al*.* (2015), and many others (Komarova 2006; Haeno et al. 2013; Jackson et al. 2014).

**Fig. 3:**
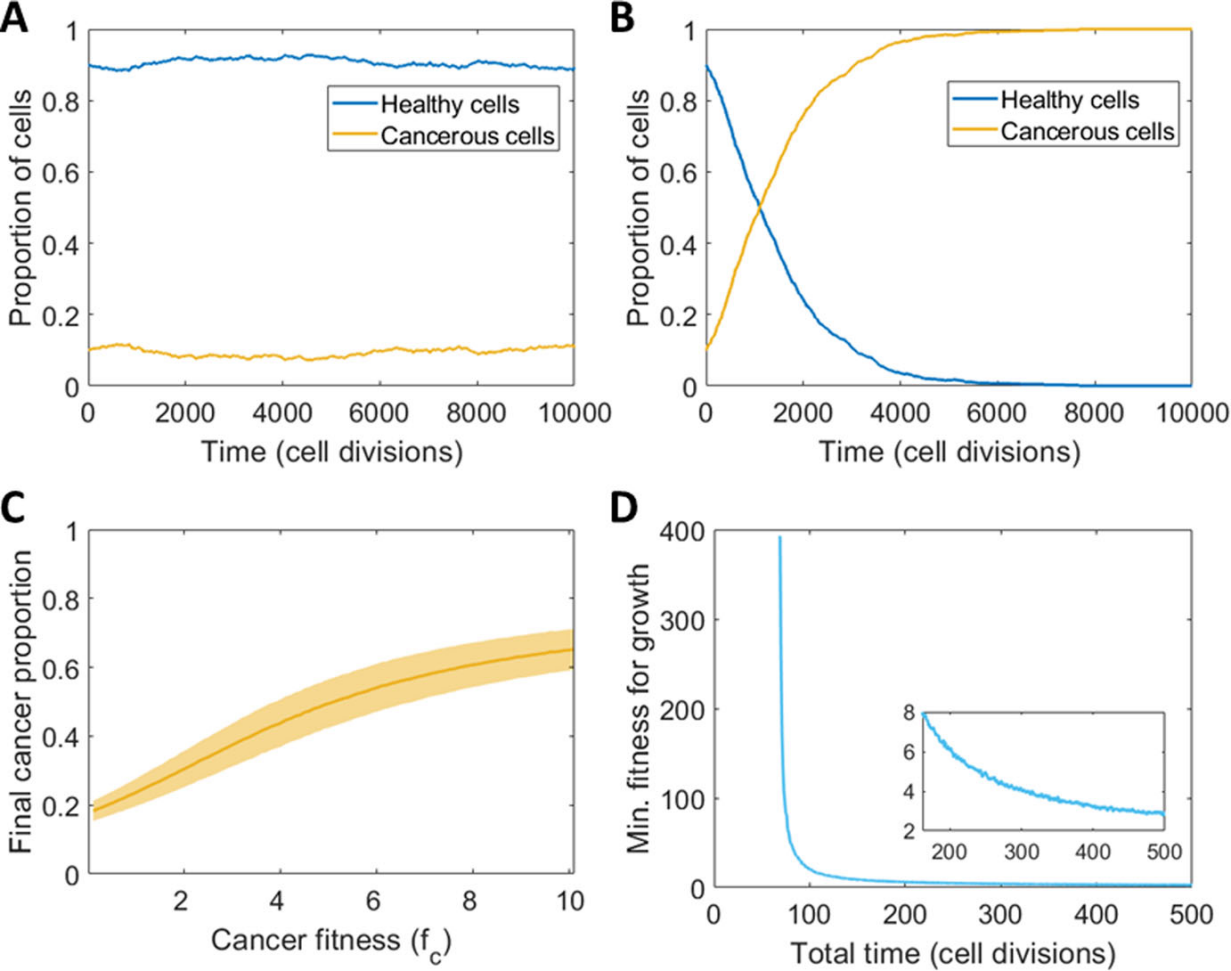
The predicted dynamics of the cancer cell-healthy cell Moran model in the absence of mutation and treatment. **A**, **B** Typical simulations of the Moran model for **A** equal fitness (${{{f}}}_{{{c}}}={{{f}}}_{{{h}}}=1$) and **B** unequal fitness (${{{f}}}_{{{c}}}=5,{{{f}}}_{{{h}}}=1$) with standard deviation of ${{{n}}}_{{{total}}}=100$ simulations in Figure S1. **C** Simulation of the Moran model for total cell divisions ${{M}}=100$ to find the final cancer proportion for different ${{{f}}}_{{{c}}}$, average and standard deviation of ${{{n}}}_{{{total}}}=1000$. **D** Required minimum cancer fitness ${{{f}}}_{{{c}}}$ for the cancer population to exceed 50% of the population, i.e. ${{{N}}}_{{{c}}}>0.5{{N}}$, where ${{N}}=100$ for varying total time, i.e. number of cell divisions ${{M}}$, and the cancer population initially only one cell (Color figure online)

Of note, if the fitnesses are equal (i.e. ${f}_{c}={f}_{h}$), then (1) and (2) are equal probabilities and it is with equal probability that cell type $c$ or cell type $h$ increases in a single timestep. Here, the probability of being selected to proliferate is proportional to the size of that cell’s type in the population, similar to the probability of being selected for removal from the population. Given this, and the fact there is a fixed total cell count $N$, we expect one cell type to fixate eventually. While the law of large numbers tells us that the average would be 50% of each cell type and we see this when we average many simulations, the cell populations won’t necessarily remain at this value given that once they reach $N$ or 0 they are fixed there for all cell divisions when mutation is not considered (Fig. 3 and Figure S2).

### Modelling Mutation and Intra-Clonal Heterogeneity

Using the simple Moran process described in Sect. 2.1, mutations from the original cancer into new cancer clones are considered. We assume there is a fixed chance that a mutant clone will emerge when the original cancer reproduces. This will be modelled with only one cancer clone to represent the growth’s genetic complexity. Our simulation does not consider back-mutation, only driver mutations towards more fit cancer types. Passenger mutations are not present in this model as equally fit clones of healthy or cancerous cells are not considered. Rather, each cell type is considered as the collection of all cells with the same fitness advantage, regardless of potential genetic differences.

For this model, mutation introduces the type $m$ cancer clone, referred to as mutated cancer, with corresponding mutation probability ${r}_{m}$ (Fig. 2B). The chance that a mutation occurs when type $c$ cells reproduce, i.e. the probability of mutation, is given by

2 $$ P\left( {m {\text{mutation}}} \right) = \frac{{r_{m} N_{c} f_{c} }}{{N_{h} f_{h} + N_{c} f_{c} + N_{m} f_{m} }}. $$

If a cancer cell undergoes mutation, it replaces the cell chosen to die by the new mutant cell type, keeping the total number of cells constant $N={N}_{c}+{N}_{h}+{N}_{m}$, where ${N}_{m}$ is the number of mutant cancer cells. This modelling formalism could naturally be extended for mutations of type ${m}_{2},\dots {m}_{n}$ cells equivalently, where their mutations can occur alongside each other. After the first driver mutation to a mutated cell type occurs, this mutation continues and their reproduction and death occur in the same way as type $c$ and $h$ cells, with fitness ${f}_{m}$.

### Modelling Treatment

Treatment can be represented in the model as a reduction of cancer fitness; either reducing their reproductive advantage over healthy cells or introducing a reproductive disadvantage. Depending on the treatment type, some drugs can be more or less effective on different cancer clones (Nikolaou et al. 2018). This is equivalent to considering a non-uniform effect of treatment on the fitness values for each clone. Typically, treatment may be targeted towards the original cancer, but it could also be targeted towards a mutant clone (Nikolaou et al. 2018).

In the model, treatment is introduced by considering some efficacy for different cancer clones, such as ${\text{eff}}_{c}$ and ${\text{eff}}_{m}$, which describes how effective treatment is for those cell types (0 having no effect and 1 being completely effective). Using these, the fitnesses of the cancer clone cells are reduced while treatment is active to give a modified fitness:

3 $$ {\text{ef}}_{c} = \left\{ {\begin{array}{*{20}l} {f_{c} \left( {1 - {\text{eff}}_{c} } \right),} \hfill & {t_{start} \le t \le t_{start} + t_{length} } \hfill \\ {f_{c} ,} \hfill & {{\text{elsewhere}}} \hfill \\ \end{array} } \right.. $$

Here, treatment is only assumed to affect the reproduction of cancerous cells, and not increase the chance of death. Specifically, a single short high dosage treatment may be considered, where the treatment efficacy remains constant within some period with a length of ${t}_{length}$ starting at ${t}_{start}$, and treatment is not active outside of the period. It may also be the case that a lower dose is injected over a larger period, which considers a lower efficacy.

Given that a drug’s presence in the body is expected to reduce after it is administered, treatment effectiveness could also reduce over time. We assumed a drug’s half-life ${t}_{1/2}$ may be used to model a waning treatment efficacy which is function of time since administration. Thus, an alternative model to Eq. (3) for treatment captures the drug efficacy decreasing over time by a decaying exponential, where it begins at the typical efficacy at the start of treatment and decays over time, as follows:

4 $$ {\text{ef}}_{c} = \left\{ {\begin{array}{*{20}l} {f_{c} \left( {1 - {\text{eff}}_{c} e^{{ - {\text{ln}}\left( 2 \right)\left( {{\text{t}} - {\text{t}}_{{{\text{start}}}} } \right)/{\text{t}}_{1/2} }} } \right),} \hfill & {t \ge t_{start} } \hfill \\ {f_{c} ,} \hfill & {t < t_{start} } \hfill \\ \end{array} } \right.. $$

See Eqs. (6)–(12) in the Appendix for a full list of transitional probabilities including those under the effect of treatment.

### Analytical Expressions of the Moran Model

Although simulations of the Moran model provide an understanding of the random nature of this process, it can sometimes be more beneficial to use an analytic expression for the expected results. Analytic expressions can reduce the computational cost previously incurred by evaluating ${n}_{total}$ simulations to achieve the average or expected result. Using the probabilities governing cell reproduction and death in Eq. (1) (1–3), the expected evolution of the cancerous cells (type $c$) over cell divisions ($n$) can be determined by the recursive sequence for the conditional expectation:

5 $$ {\text{E }}\left[ {N_{{c_{n} }} |N_{{c_{n - 1} }} } \right] = N_{{c_{n - 1} }} + \frac{{N_{{c_{n - 1} }} f_{c} }}{{N_{{c_{n - 1} }} \left( {f_{c} - f_{h} } \right) + Nf_{h} }} - \frac{{N_{{c_{n - 1} }} }}{N},{ } $$

given some initial cell count ${N}_{{C}_{0}}.$ This definition holds similarly for the expected evolution of healthy cells (type $h$). This model closely matches the average Moran simulation when the initial number of cells is higher than $\sim 10\%$ of $N$. Below this threshold, the average simulation is affected by trajectories where the cancerous cell type dies out due to stochastic fluctuations. This produces an average growth that is not expected of a typical simulation and does not match Eq. (5). Mutation can be introduced into Eq. (5) by applying the mutation probability (Eq. (2)) to develop a recursive model for the expected evolution of mutated cancer cells (type $m$). Treatment can be introduced by allowing fitness to be varied as in Eq. (3) with constant efficacy, or Eq. (4) with treatment efficacy decreasing over time. In this work, we are motivated to obtain a computable expression that leads to ballpark estimates of the proportion of cells of each type similar to work by Dinh et al*.* (2020).

## Experimental Measurements of Tumour Growth

In this work, two sets of tumour growth measurements were used to validate and calibrate the model. The efficacy of Herceptin was measured by Lewis Phillips et al*.* using an in vivo human breast cancer mouse model (Lewis Phillips et al. 2008). Tumour volume was measured in 6–10 mice with and without administration of Herceptin (tratuzumab). KPL-4 human breast tumour cells were inoculated (3 million cells per mouse) into the mammary fat pads of SCID beige mice. Injections of tratuzumab were given once every week for 4 weeks for four total injections in the treatment group. Tumour size was monitored twice weekly using caliper measurements. Numerical simulations of the model and parameter estimation.

Details for the simulation pipeline of our Moran model can be found in Algorithm 1 and Figure S1. Numerical simulations of the Moran model are performed using MATLAB (R2023b). A summary for the ranges of parameters and their meaning is given in Table S1. When estimating parameters from data, the analytic recursive model in Eq. (5) is used for consistency and computational efficiency to estimate parameters. The Moran model is then simulated with the obtained parameter estimates and the mean of 100 simulations is confirmed to match the data. In cases where the initial number of cells are not relatively low, the average of 100 simulations matches the expected result closely within the data range. Other processes for fitting parameters have been demonstrated by West et al*.* (2016b) who fit parameters in their Moran model by implementing least-squares fitting. Dinh et al*.* (2020) use the conditional expectation of their Moran model to estimate the selection coefficient and mutation rate from data. There are other inference methods, such as Approximate Bayesian Computation (ABC), that have also been used to estimate parameters in a Moran model (Heyde et al. 2021; Lynch et al. 2022; Beerenwinkel et al. 2015; Rabosky 2009).

In the case study considered in Sect. 3.4, the ratio of fitness between cancerous and healthy cells (i.e. ${f}_{c}/{f}_{h}$ or ${f}_{m}/{f}_{h}$) is fitted to data, alongside other measures such as mutation rate ${r}_{m}$, and treatment efficacies. The complete expected Moran simulation is calculated using the recursive model for different combinations of these parameters and the Root Mean Square Error (RMSE) between this result and the data is recorded. For each complete simulation, the parameters that gave this error is recorded if the error is lower than the error given by the previous optimal parameters. Once all combinations are used to generate Moran simulations, the combination of parameters that provides the lowest error is taken to be the optimal fit to the data. The general complete process for the data fitting is shown in Algorithm 2 in the Appendix. Here, values for the fitness were checked between 1 and 2 with a step size of 0.001.

The fitness ratios ${f}_{c}{/f}_{h}$ and mutation probability ${r}_{m}$ is first fit to the vehicle data, and then the treatment efficacies ${{\text{eff}}}_{{\text{c}}}$ and ${{\text{eff}}}_{{\text{m}}}$ is fit to the data depicting growth under treatment, using the determined fitness ratios and mutation probability. Here, other information about the data is estimated, such as the total number of cells, $N$, and the amount of cell divisions per hour. $N$ is estimated by examining the growth shown in the data and visually extrapolating the total number of cells, and the cell divisions per hour are estimated to be $N/8$ as Ehrlich cells (those considered in the data) take eight hours to divide on average (Kroll et al. 1987). Given that Ehrlich cells divide around every 8 h (Kroll et al. 1987), it can be assumed for this model that the amount of time per simulated cell division is $8/N$. Additionally, this cancer may be treated with Herceptin, which is found to have an average half-life of 5.8 days (Goldenberg 1999; Bruno et al. 2005). Simulations were run for treatment efficacies between 0 and 100% with a step size of 0.1%.

Code is available on Github https://github.com/AdrianneJennerQUT/Impact-of-resistance-on-therapeutic-design

## Results

### Prediction for Cancer Evolution Under Two Cell Moran Model

To qualitatively confirm the Moran model’s ability to capture the growth of a population of cancerous cells, we simulate the model with no mutation $$({r}_{m}=0$$) starting with 10% cancer cells. A typical example of this is seen in Fig. 3A, with the average and standard deviations shown in Figure S2A. When the fitness of both cell types is equal ($${f}_{c}={f}_{h}=1)$$, as expected, the percentage of type $$c$$ cells remains around its initial value in the short term, however, for sufficiently long simulations, we expect one population to fixate given we have a finite population size, as seen in Figure S2D and by the expanding standard deviations in Figure S2A. If type $$c$$ cells are given an advantage in the form of a higher fitness, i.e. $${f}_{c}=5$$ and $${f}_{h}=1$$, the percentage of cells increases logistically (Fig. 3B), and there is much less deviation between simulations as seen in Figure S2B. It is expected that for any $${f}_{c}>{f}_{h}$$, the cancerous cell type $$c$$ will reach fixation over time, but this will only be observed in simulations if the total number of cell divisions, $$M$$, is large enough. For example, fixing the number of cell divisions to $$M=100$$ and varying the fitness, we see that the average cancer population ($${n}_{total}=1000$$ simulations) does not reach fixation in this time (Fig. 3C), despite a tenfold fitness advantage (see Figure S2C for the effect of the choice of $${n}_{total}$$ on the average simulation value). We sought to understand further the influence of the total number of cell divisions on the ability of the cancer population to grow (Figure S1E). We see that the average cancer population only reaches fixation or a high proportion of the total cell population if the fitness value is high and the number of cell divisions is also sufficiently high. This suggests that cancer cell fitness can be quite low, and a cancer will still evolve if it is given sufficient time to take over the healthy cell population. We quantified the minimum fitness value for a given number of cell divisions for the tumour population to reach over 50% of $$N$$, starting from initially 1 cell (Fig. 3D). We found there is a clear asymptote at around 67 cell divisions, which implies that it is impossible for cancerous cells to reach 50% given less than 67 cell divisions for this value of $$N$$. This is unsurprising, as the cancerous population starts with only one cell and are only able to increase by one cell at each time step, with a higher chance of death as the cancer population increases. As such, there must be some required time (in this case $$0.5N$$ or larger) before these cells can reach a given threshold.

### Evaluation of Treatment Efficacy Under Cancer Cell Mutation Moran Model

**Fig. 4:**
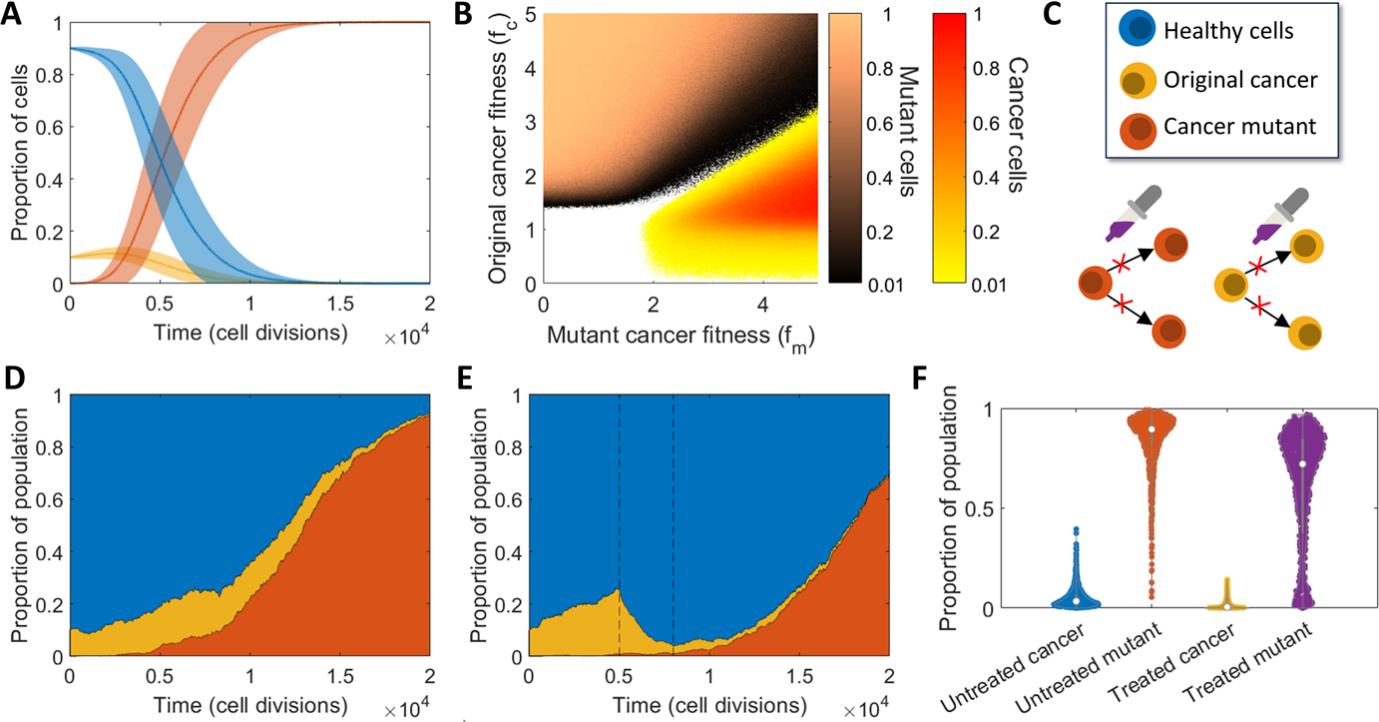
The impact of mutations on cancer growth and treatment. **A** Simulation of the Moran model showing tunneling of the mutant cancer, under high mutant fitness (${f}_{c}=1.1{f}_{h}$, ${f}_{m}=3{f}_{h}$), average and standard deviation of ${n}_{total}=1000$. **B** Regions depicting the most likely cancer type to fixate under varying ${f}_{c}$ and ${f}_{m}$. The proportion of simulations (${n}_{total}=100$) where either the original cancer cells or mutant cancer cells fixated is plotted with corresponding colour bars. The brown colour bar gives the proportion of simulations where mutant cells fixated and the orange to yellow colour bar gives the proportion of simulations where cancer cells fixated. White regions represent parameter combinations where neither cancer cell types fixated in any of the simulations, an example of this is shown in Figure S3D. **C** Model schematic depicting the effect of treatment in blocking or reducing the likelihood of cancer and mutant type proliferation. **D**, **E** Typical simulations depicting relative area of cell types over time with the inclusion of a fit mutant clone under **D** no treatment and **E** single high dosage treatment active for 3000 cell divisions. The period for which treatment is active is denoted by dashed black lines. **F** Comparison of the final proportions of both cancer types (cancer and mutant) without treatment and under single high dosage treatment for ${n}_{total}=1000$ simulations. Presented as violin plots overlayed with individual data points (Color figure online)

Moran models are regularly used to track the probability of tunneling, which occurs when a cancer mutant reaches fixation before the original cancer (Komarova 2006; Ashcroft et al. 2015). Komarova (2006), Haeno et al*.* (2013) and Jackson et al*.* (2014) each consider a Moran model with three cell types, where the third type is a mutation from the original cancer with higher fitness. Each of these models discover that tunneling occurs naturally at some rate depending on the fitnesses of the cancerous and mutated cells. Using our model with parameters $N=1000$, $M=\mathrm{20,000},{f}_{h}=1$, ${f}_{c}=1.1$, ${f}_{m}=1.5$, ${r}_{m}=0.01$, there is around a 0% chance, of tunneling, but if ${f}_{m}=3$ (i.e. the fitness of the mutant clone increases) then there is a 96.5% chance of tunneling. The average simulation (${n}_{total}=1000$) under these conditions is in Fig. 4A where the higher fitness is considered.

To quantify the likelihood of tunneling given varying cancer cell and mutant fitnesses, we have coloured the region of our $({f}_{m}, {f}_{c}$) parameter space to denote which parameter combinations give rise to tunneling of cancer cells or mutant cancer cells, seen in Fig. 4B. For a fixed number of cell divisions $M$ and total simulations ${n}_{total}$ the proportion of simulations giving rise to tunnelling for either mutant or cancer cells is depicted by shaded colours. A $\left({f}_{m},{f}_{c}\right)$ combination was considered to give rise to tunneling of a particular cell type if that cell type reached fixation and the proportion of the total simulations ${n}_{total}$ that gave rise to tunneling were higher for one cell type over another. Areas of white space represent (${f}_{m}, {f}_{c}$) combinations that did not give rise to either mutant or cancer cells fixating in the fixed number of cell divisions $M$ (for example, see Figure S2C). We see there are clear regions of the $({f}_{m}, {f}_{c})$ parameter space that give rise to one cell type outperforming the other. In particular for ${f}_{m}>2$ and ${f}_{c}>1.5$ there is a ratio of ${f}_{c}/{f}_{m}\approx 0.6,$ either side of which one cancer cell type wins. For example, ${f}_{c}>0.6{f}_{m}$ results in the original cancer outperforming the mutant. Largely, this image is also affected by the probability of mutation occurring, ${r}_{m}$, which is fixed at 0.01.

To investigate the impact of mutation on cancer growth under treatment, we simulated the cancer cell model with a single mutated clone. Using the choice of parameters above, a typical Moran process is simulated and displayed in Fig. 4D, alongside a similar simulation with treatment applied in Fig. 4E. Here, treatment begins at 5000 cell divisions and ends at 8000 cell divisions, with 80% efficacy against the original cancerous cells (type $c$) and 40% efficacy against mutant cells (type $m$). We find that the mutant clone will take over even in the presence of treatment if it is present when treatment begins. Given that the conditions are such that tunneling of the mutant clone is expected to occur, it is also expected that treatment may fail to control the mutant clone. As a result, the tumour’s response to the treatment may also be examined to determine if tunneling is occurring with a resistance cancer clone. Further examples of these behaviours may be seen in Figures S3A and S3D. Using these parameters, the final proportions of cancer types $c$ and $m$ were recorded and examined with and without treatment as seen in Fig. 4F. Here it can be seen that treatment reduces the final proportions of each cancer type, though there remains a high possibility that the mutant clone approaches fixation with large variation in the final mutant number, suggesting that in some instances, the healthy cells may regain control.

### Evaluation of Treatment Strategies

**Fig. 5:**
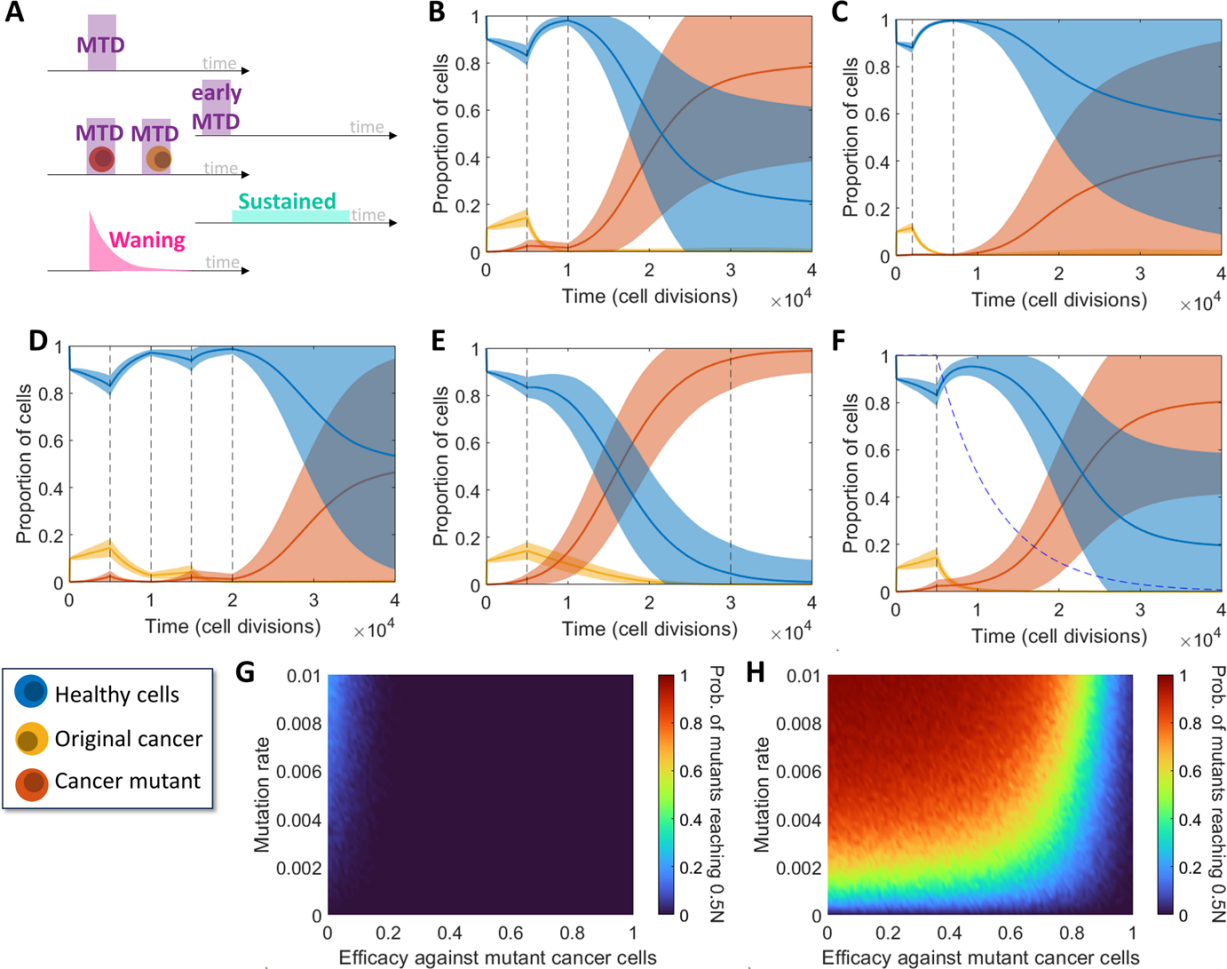
Comparisons between different treatment methods and models. **A** Descriptions of the five treatment methods considered in (**B**–**F**): a MTD, an early MTD, a dual dosage (one targeting the mutant followed by one targeting the cancer), a sustained low dose and a waning treatment. **B**–**F** Simulations of cancer growth under varying treatment strategies, average and standard deviation of ${n}_{total}=1000$. **B**, **C** Single high dosages applied for ${t}_{length}=5000$ cell divisions between the vertical dashed lines **B** after the mutant clone is expected to arise and **C** before the mutant clone is expected to arise, i.e. early treatment. **D** Dual dosing with the first targeting the mutant cancer clone and the second targeting the original cancer clone. **E** Sustained dosing with an efficacy one fifth of that in other tests. **F** High dosage with a waning drug concentration modelled by the blue dashed line. **G**, **H** Proportion of simulations (here ${f}_{m}=1.6$ and $M=\mathrm{20,000}$) with varying ${r}_{m}$ and ${\text{eff}}_{m}$ where mutant cells reach a population of $0.5N$ under **G** single high dosage treatment, and **H** sustained treatment with efficacy scaled by 1/3 and length scaled by 5. Corresponding two parameter plots for the original cancer are given in Figure S3D (Color figure online)

Using the Moran model with three cell types (healthy $h,$ cancerous $c$, and mutant $m$), different treatment strategies were tested and compared (Fig. 5A): applying the maximum tolerated dose (MTD) after mutant growths arise, earlier dosing, dual dosing, sustained dosing, and waning treatment. Here, the MTD is assumed for all simulations except the sustained dosage and waning treatment. The sustained dosage is a reduced dosage (lower treatment efficacy) that is applied for a longer period, and the waning treatment is an exponential decrease of the effect of treatment to capture pharmacokinetic drug dynamics. The dual dosing treatment considered that the mutant cancer was targeted in the first dose and that the second dose targeted the original cancer. See Fig. 5B–F for the mean and standard deviation of 1000 model simulations.

In general, the presence of a mutated cancer clone provides the cancer growth with an inherent treatment resistance which makes treatment difficult. In certain cases, particularly the MTD, sustained treatment and waning treatment, the presence of the drug only aided the growth of the mutated clone by reducing the original cancer clone. As such, applying the MTD can be effective assuming that the treatment is effective towards the mutated clone or if the treatment dose is high enough, but can fail to eliminate the mutated cancer otherwise (see Fig. 5B). Sustained treatment often fails similarly since the reduced dosage is not strong enough to eliminate the mutated cancer (see Fig. 5E).

This motivates the use of early dosing which tends to eliminate the cancer before any mutation arises and does not require a large dosage if targeted towards the original cancer, as seen in Fig. 5C. Finally, dual dosing was considered to target the mutated cancer in a first dosage to reduce or eliminate it, and then apply a second dose targeted towards the original clone. This provided varying results depending on mutation (see Fig. 5D), but it is again determined that intra-clonal heterogeneity presents challenging resistances to treatment.

An alternative treatment model considering a waning effect on the drug’s concentration is considered in Fig. 5F, where the concentration is modelled by a decaying exponential based on the drug’s half-life. In this case, the half-life is arbitrarily selected to be 5000 cell divisions, though this may be more appropriately selected for specific cases. Here, the waning treatment model represents a more natural transition between active and ineffective treatment, in this case showing results less promising than consistently administering the MTD, and more promising than administering a consistent sustained dose. Individual model trajectories for the five treatment scenarios are plotted in Figure S4 and corresponding violin and scatter plots for the final proportion of healthy, cancer and mutant cells is given in Figure S5–S6. Figure 5G, H compare the mutant cancer (type $m$) responses to treatment with the MTD and sustained treatment respectively, where the mutation rate and efficacy against mutant cells vary. These plots consider the proportion of simulations where mutant cells grow to at least $50\%$ of the total cell population $N$ within $M=\mathrm{20,000}$ cell divisions. Additionally in Figures S3D, a similar comparison is made for the original cancer cell type (type $c$). For the selected parameters, the original cancer (cell type $c$) will almost never reach $50\%$ of $N$, which is likely due to tunneling rather than treatment, as the mutated cancer is expected to take over quickly. Figure 5G, H show that the mutated cancer cell clone (type $m$) is more likely to grow to a substantial size in the sustained treatment scenario if the mutation rate is high and treatment is not as effective against that cell type, as expected. Specifically using the MTD in this case, low efficacy is required for the mutated cancer to have a non-zero chance of reaching $50\%$ of $N$. It is evident that treatment with the MTD is overall more effective than sustained treatment when changes in efficacy and mutation rate are considered. Some selections of these parameters can facilitate effective sustained treatment such as zero mutation rate or a treatment efficacy of $100\%$, however these selections are not practical.

### Herceptin Case Study

**Fig. 6:**
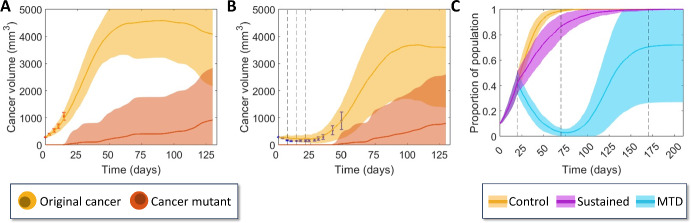
Summary of model fitting to experimental data and testing. Comparison between data and model fits for vehicle cancer growth data (**A**) and growth under Herceptin treatments applied four times (**B**) (Lewis Phillips, et al. 2008) starting at zero days and re-administered every seven days with waning drug concentration, simulations shown with mean and standard deviation over ${n}_{total}=100$. Parameter values and RMSE plots are in Table S2 and Figure S8. **C** Comparison between treatment techniques with waning drug effects using the model fit to the data in (**A**, **B**), with a single high dosage starting at 20 days and lasting for 50 days (period within first two black dashed lines), and a sustained dosage starting at 20 days with efficacy scaled by 1/3 and length scaled by 3 (i.e. until the final dashed line). Note that for all simulations the total population size remains constant $N={N}_{c}+{N}_{h}+{N}_{m}$, where it is assumed initially that healthy cells are present in the model near confluence initially (Color figure online)

To validate the proposed model and the findings in Fig. 5, model parameters were fit to measurements for the growth of HER2-Postive breast tumours in mice, with and without Herceptin treatment, (Lewis Phillips et al. 2008) (see Sect. 2.5 for more details). The fitness ratios for cancer and mutants to healthy cells were fit from vehicle measurements (Fig. 6A and Figure S8A). The minimum RMSE returned a fitness of around ${f}_{c}=1.033{f}_{h}$ for cancer cells, ${f}_{m}=1.289{f}_{h}$ for mutant cells, and ${r}_{m}=1.1\times {10}^{-6}$. This mutation probability matches ranges obtained by others in the literature with values seen between ${10}^{-9}$ and ${10}^{-5}$ (Cassidy and Craig 2019; Iwasa et al. 2566; Bozic and Nowak 2014; Coldman and Goldie 1983).

While a minimum in the RMSE is possible, there is a clear relationship between the mutation probability and the fitness ratio of mutant cells to healthy cells suggesting the mutation framework is not practically identifiable from this data set. Fixing the cancer fitness and mutant fitness ratios, alongside the information about the half-life of Herceptin and the dosages indicated by Lewis Phillips et al*.* (Lewis Phillips et al. 2008) allows the recursive model to the fit to treatment data, assuming a waning effect (Eq. (5)), also seen in Fig. 6B. It is found here that this treatment is around 5% effective against this cancer growth (i.e. ${\text{eff}}_{c}=0.052$). Given the small mutational probability, the mutant cells are not present early enough for the efficacy of the treatment on the mutant cells to have any certainty in its estimate from the treatment measurements. Fitting ${f}_{c}/{f}_{h}$ with ${r}_{m}=0$ gives a qualitatively similar fit in both scenarios (Figure S7).

We then considered these parameters in our previous treatment scenarios from Fig. 5A, however, particularly focussing on MTD. Results shown in Fig. 6C compare a single high-dose injection (MTD) to a sustained low-dose injection, both with waning drug concentration after treatment concludes. It is seen from the typical simulations that this sustained treatment with Herceptin does not have a strong impact on the cancer’s growth, though the single high-dose treatment is able to almost eradicate the cancer entirely, however, regrowth eventually occurs.

## Discussion

Moran models present a useful mathematical tool that can be used to capture the occurrence of resistant-mutant clones in the cancer population and the impact this has on cancer therapy. Resistance is one of the major causes of cancer related deaths, and understanding how the presence of resistance cancer subpopulations impact new therapeutic designs is crucial to the drug development pipeline. In this work, we develop a simple stochastic model for cancer proliferation and mutation using a Moran process. With this model, we investigate the impact of therapeutic design on treatment efficacy focusing on two main treatment protocols: short treatment windows with the MTD administered and longer treatment windows with lower treatment dosages. These are referred to as MTD and sustained treatment. We then examined how a model such as this could be used to match to experimental data.

Using a simple two cell (cancer and healthy cell) Moran model, we replicated the biological behaviour expected of a non-cancerous and cancerous subpopulation, whereby, cells with a fitness advantage grow logistically. Most interestingly, we quantified the minimum fitness needed for a given number of cell divisions to result in the cancer growing to more than 50% of the population. We found that for a fixed number of cells (i.e. 100 cells), as the number of cell divisions increased, the minimum fitness needed to grow the tumour decreases exponentially. There was a clear asymptote providing the minimum number of cell divisions needed for the tumour to grow beyond 50% of the population. This asymptote is intuitive and represents the minimum number of timesteps needed to increase the tumour population from 1 to 50. Introducing mutations, we were then able to quantify the regions of the mutation parameter space that would provide successful tunnelling for the mutant population, i.e. ${f}_{c}/{f}_{m}<0.6$.

Investigating the effects of a MTD treatment, simulations first revealed the large variation in responses to treatment of the mutant population. For some trajectories, there were no mutants and minimum cancer cells, suggesting the healthy cell population had regained control (Figure S5 and Figure S6). In particular, we notice that in cases where mutant cells do not regain control after treatment, the model proposes for some non-zero proportion of simulations, healthy cells can regrow and become 100% of the cell population. This is most clearly seen in Early MTD and Dual MTD where > 45% of the simulations suggest healthy cells can regrow (Figure S6). Furthermore, the final average mutant population is decreased in the presence of treatment. Comparing the MTD to sustained treatment, it was clear that MTD is more likely to be effective than the sustained treatment for a range of cell fitness, i.e. cancer growth rates. This is an important finding given the interest in using sustained dosages and suggests that more work is needed to investigate the impact resistance may have on this treatment.

We calibrated the Moran model to in vivo control and breast cancer treatment data using our recursive form of the Moran model. We found that in this parameter regime our results were corroborated with MTD being most effective and sustained treatment being virtually ineffective. There are more robust methods for evaluating likelihoods for stochastic birth–death processes that, with a more detailed dataset, could have been used to improve the parameter estimated from the in vitro and in vivo datasets (Boskova et al. 2014; Crawford et al. 2018) particularly given the practical identifiability challenges presented with the vehicle and treatment data. Furthermore, likelihood-free methods, such as ABC (Lynch et al. 2022), may also have provided more accurate estimates for the parameter values and the uncertainty in their estimation. As noticed by Komarova (2006), spatial models can be more accurate at predicting mutation parameter compared to non-spatial models like ours. Future work will look to combine the simple Moran model presented here with more detailed transcriptomic data that may be fit using these well-established methods.

We found that early treatment is more effective than later treatment, which is unsurprising given that early administration reduces the evolutionary process of cancer mutation and, hence, the tumour’s genetic complexity. This finding could be an artifact of the choice of modelling cancer evolution with a Moran model, essentially a homogeneous Markov process, and therefore requiring less effort to reduce the population of cancer cells when the population size is already small, i.e. early in the simulations. West et al*.* (2016a) suggested that treatment should be administered in the early stages to prevent genetic complexity. In addition to early treatment, we also found dual dosing of treatment was more effective than other treatments, such as waning treatment. This suggests that targeting the more fit mutant clone may be more beneficial, though large variation was seen in our results. In the past decade, multiple clinical trials have investigated the use of intermittent therapy, which applied on/off treatment cycles after an initial induction period (Chahoud 2023). These therapies exploit the assumption that intermittent treatment strategies exploit evolutionary principles to delay the onset of resistance while decreasing accumulated drug doses and reducing toxicity (Chahoud 2023). These therapies have mixed results (Chahoud 2023).

Waning treatment was found to be just as ineffective as sustained treatment, suggesting that the waning of treatment efficacy due to pharmacokinetics might be what causes issues with efficacy. Furthermore, this suggests that the waning treatment (if more representative of true human dynamics) should be what we compare to sustained treatment and comparing the violin plots for the final amount we see there is more variation in the sustained treatment then the waning treatment—suggesting some people might response positively to sustained treatment.

There are limitations to our current modelling framework and future work can look to improve and also further validate experimentally the predictions of the model. For example, we do not consider a cost associated with resistance to therapy, and it has been suggested that if sensitive populations are much larger than the resistant population in a cancer, it usually indicates that there is a high fitness cost for resistance (Chahoud 2023). Fortunately, the ratio of sensitive-to-resistant cells can often be inferred from the initial response to therapy (Chahoud 2023) and future work hopes to use informed initialisation of the model. In addition, we are using a non-spatial model of cancer cell behaviour to capture what is often a very spatial process. Given that our model is sufficiently simple and does not try to link proliferation and death to real tumour processes, it can be thought to capture a range of biological phenomena occurring in the tumour.

In this work, we present a simple Moran framework that can be used to quantify the impact of resistance on a range of treatment modalities. Most significantly, we find that early treatment is the most effective form of treatment, followed closely by dual treatment where the mutant clone and then the cancer clone are targeted in succession. MTD is an effective therapy, when compared to sustained dosages and this is most seen most clearly in the case where the model was matched to experiment measurement for breast cancer treatment with Herceptin. This study suggests more work needs to be done to investigate sustained dosage therapies in clonal heterogeneous subpopulations to investigate whether resistance is going to hinder this treatment’s effectiveness.

### Supplementary Information

Below is the link to the electronic supplementary material.

Supplementary file1 (PDF 1235 kb)
