# Supplementary material for: Impact of Resistance on Therapeutic Design: A Moran Model of Cancer Growth
Source: Bull Math Biol. 2024 Mar 19;86(4):43. doi: 10.1007/s11538-024-01272-6 (PMC10950993; doi:10.1007/s11538-024-01272-6)
Supplement: Supplementary file 1 — Supplementary file1 (PDF 1235 kb) [file 11538_2024_1272_MOESM1_ESM.pdf]

<u>Supplementary Information</u>: Impact of resistance on therapeutic design: a Moran model of cancer growth

Mason S. Lacy[1], Adrianne L. Jenner[1]

[1]School of Mathematical Sciences, Queensland University of Technology, Brisbane, QLD, Australia

Contents:

- Algorithm 1-2
- Full list of model equations
- Figure S1-S8
- Table S1-S2

1. Supplementary Information

Algorithm 1 Simulating Moran model with treatment and mutations

Initialise $N_c, N_m,$ and N_h
for $n = 1$ to M **do**
  $t = n\Delta t$
  if $t_{start} \le t \le t_{start} + t_{length}$
    $\mathrm{ef_c} = f_c(1 - \mathrm{eff_c})$
  else
    $\mathrm{ef_c} = f_c$
  end
  sample $r \sim U(0,1)$
  if $r < \frac{N_h f_h}{N_h f_h + N_c ef_c + N_m \mathrm{ef}_m}$ **do**
    $N_h = N_h + 1$
  else if $r < \frac{N_h f_h + N_c \mathrm{ef}_c (1 - r_m)}{N_h f_h + N_c ef_c + N_m \mathrm{ef}_m}$ **do**
    $N_c = N_c + 1$
  else if $r < \frac{N_h f_h + N_c \mathrm{ef}_c}{N_h f_h + N_c ef_c + N_m \mathrm{ef}_m}$ **do**
    $N_m = N_m + 1$
  else
    $N_m = N_m + 1$
  end
  sample $r \sim U(0,1)$
  if $r < \frac{N_h}{N}$ **do**
    $N_h = N_h - 1$
  else if $r < \frac{N_h + N_c}{N}$ **do**
    $N_c = N_c - 1$
  else
    $N_m = N_m - 1$
  end
end

Algorithm 2 Fitting M/t and f_c/f_h to vehicle data

Load time (t) and cancer cell population (p) data
Initialise $N = 1000, N_{c_0} = 1$, and $e_{best} = \infty$
for fitness ratio, $f_c/f_h = 1$ to 2 **do**
  Generate complete expected Moran simulation, N_c
  Calculate error, $e = \sqrt{avg((p - N_c)^2)}$
  if $e < e_{best}$, new best parameters are found **then**
    $f_{c_{best}}/f_h = f_c/f_h$
    $e_{best} = e$
  end
end

1.1. Full list of model transition probabilities: healthy cells, cancer cells, mutant cancer cells and treatment

The probability of a cell of type h, c or m dying respectively is given by:

$$P(\text{type } h \text{ dies}) = \frac{N_h}{N}, \tag{6}$$

$$P(\text{type c dies}) = \frac{N_c}{N}, \tag{7}$$

$$P(\text{type m dies}) = \frac{N_m}{N}. \tag{8}$$

The probabilities of cells of type h, c or m reproducing under the effects of treatment (see **Eq. (3)-(4)**) are:

$$P(\text{type } h \text{ reproduces another type } h) = \frac{N_h f_h}{N_h f_h + N_c e f_c + N_m \mathrm{ef}_m}, \tag{9}$$

$$P(\text{type } c \text{ reproduces another type } c) = \frac{N_c \mathrm{ef}_c (1 - r_m)}{N_h f_h + N_c e f_c + N_m \mathrm{ef}_m}, \tag{10}$$

$$P(\text{type } c \text{ reproduces and mutates to type } m) = \frac{N_c \mathrm{ef}_c r_m}{N_h f_h + N_c e f_c + N_m \mathrm{ef}_m}, \tag{11}$$

$$P(\text{type } m \text{ reproduces another type } m) = \frac{N_m \mathrm{ef}_m}{N_h f_h + N_c e f_c + N_m \mathrm{ef}_m}. \tag{12}$$

1.2. Supplementary figures and tables

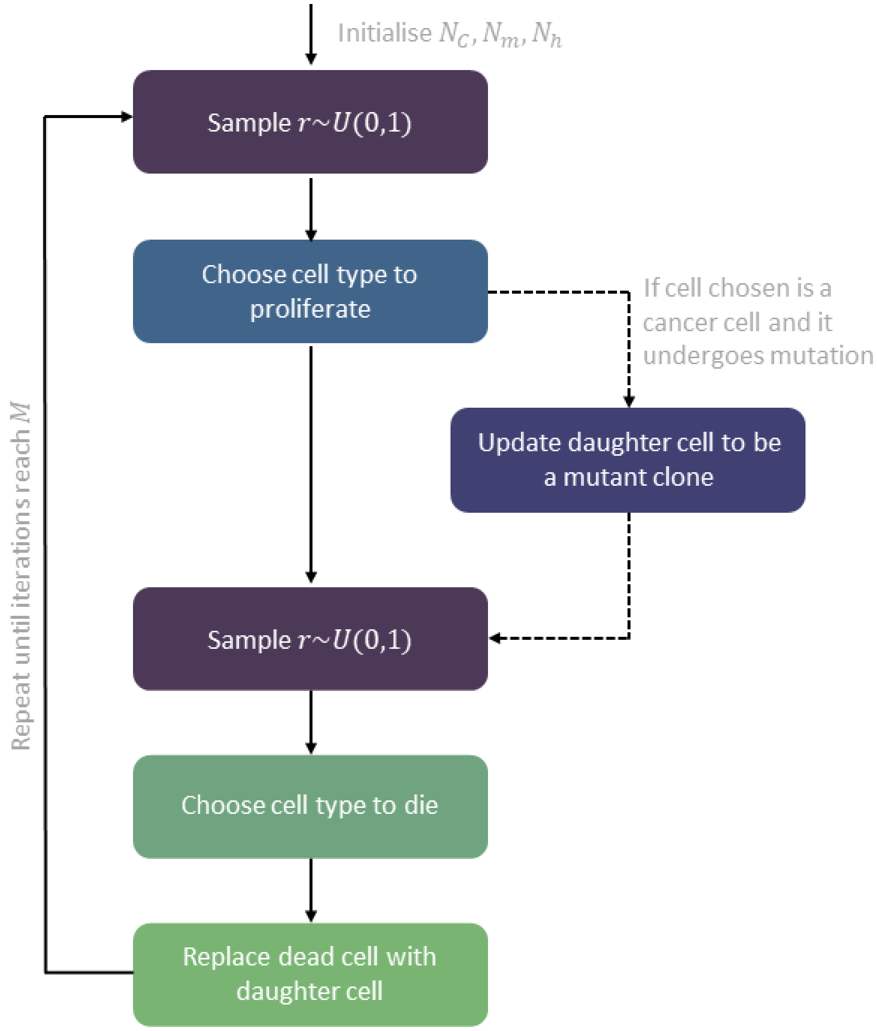

Figure S1 Moran model simulation pipeline. Schematic depicting the Moran model simulation pipeline. An initial population size for each cell type N_c, N_m and N_h is chosen and then in each step, a cell is chosen to proliferate and die based on the probabilities detailed in **Eq (1)-(4)** in the main text and **Eq (6)-(12)** in the SI. The cell created through proliferation, i.e. the daughter cell will replace the cell chosen to die, conserving the total cell population $N = N_c + N_h + N_m$. If a cancer cell is chosen to proliferate, and undergoes mutation, the daughter cell will be a mutant cell type.

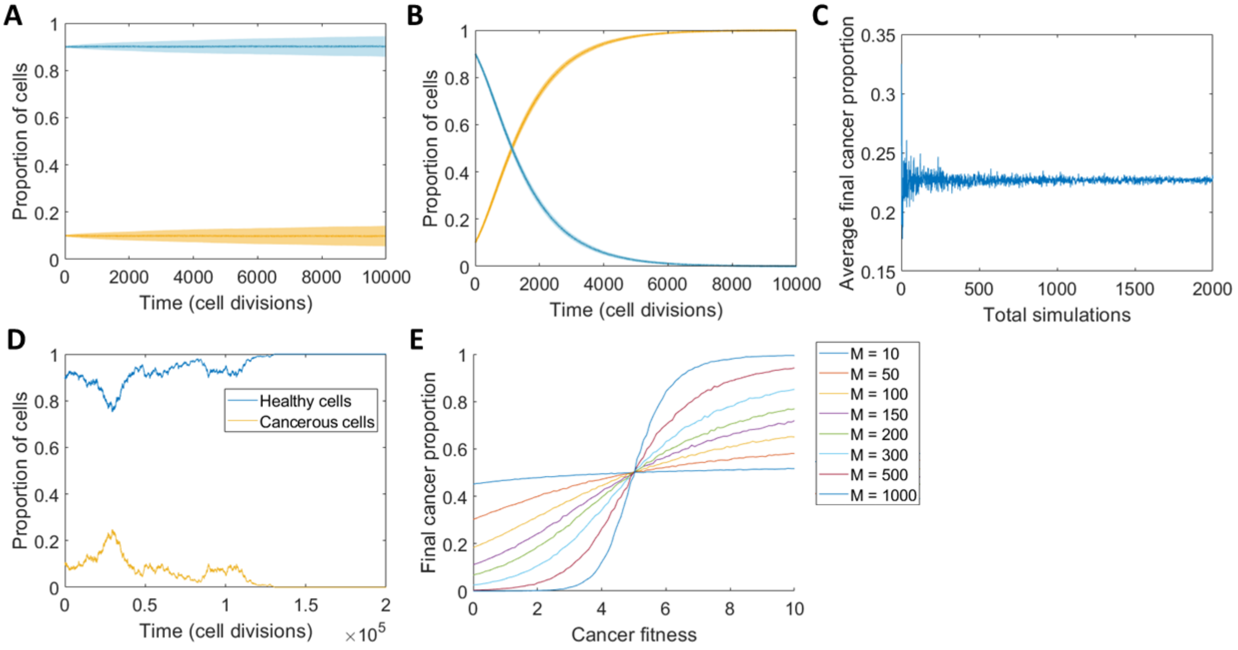

Figure S2 Moran model outputs for cancer growth in the absence of mutant clones and treatment. (A, B) Simulations of the Moran model for (A) equal fitness ($f_c = f_h = 1$) and (B) unequal fitness ($f_c = 5, f_h = 1$), with mean and standard deviation for $n_{total} = 1000$ and an initial proportion of cancer cells (yellow) or 10% and healthy cells (blue) 90%. (C) To assess the convergence of the stochastic process the average final proportion of cancer cells under a typical simulation is given as n_{total} increases. (D) Typical simulation over large time for equal fitness ($f_c = f_h = 1$), i.e. corresponding to (A) and (B). (E) Comparison between the final proportion of cancer cells as fitness varies for $M \in [10,1000]$, where M is the total number of cell divisions.

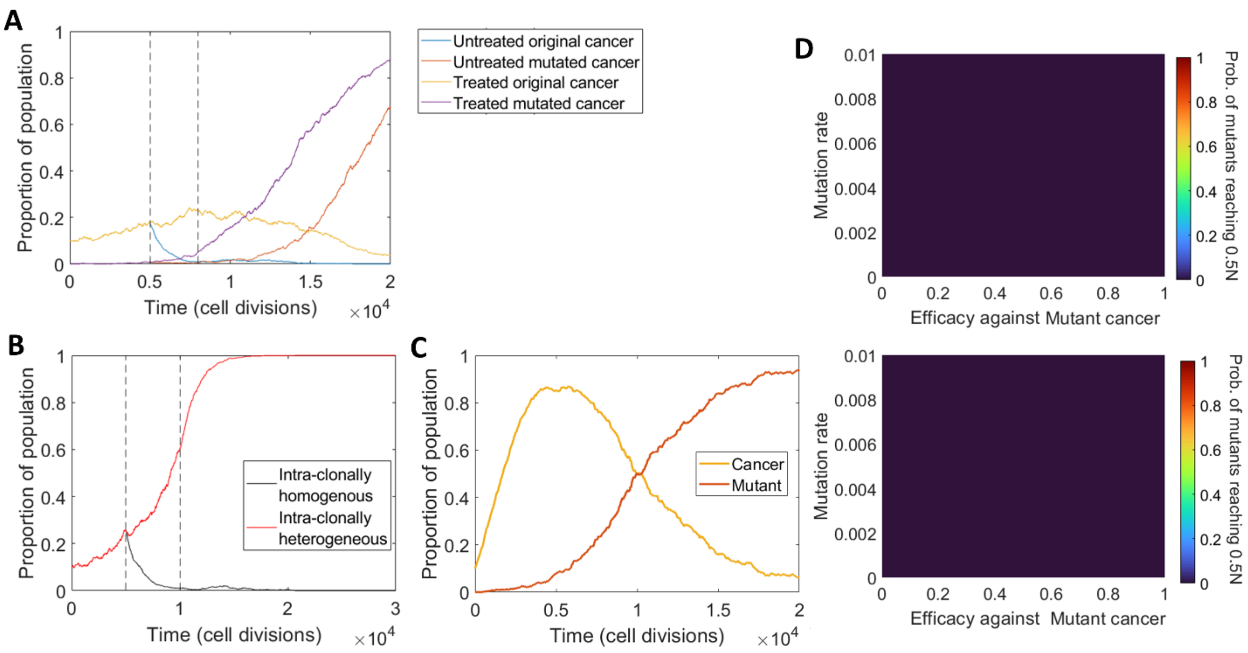

Figure S3 Supplementary figures summarising the impacts of mutant clones on cancer growth and treatment. (A) Typical simulation of intra-clonal heterogeneous cancer growth under single high dosage treatment. (B) Comparison between the effects of treatment on a cancer growth with and without the presence of a mutant clone, given typical simulations. (C) An illustrative example of what the white region in Figure 4B represents. In these regions neither cell type has fixated, but it could have been close to fixation and in an infinite time we might expect it to fixate. Parameters used were $f_c/f_h = 3.2$, $f_m/f_h = 4.5$ and the rest were the same as **Figure 4B**, i.e. $N = 1000, M = 20000, r_m = 1/1000, N_{c_0} = 100$. (D) Proportion of simulations with varying r_m and eff_m where original cancer cells reach a population of $0.5N$ under (top) single high dosage treatment, and (bottom) sustained treatment with efficacy scaled by 1/3 and length scaled by 5.

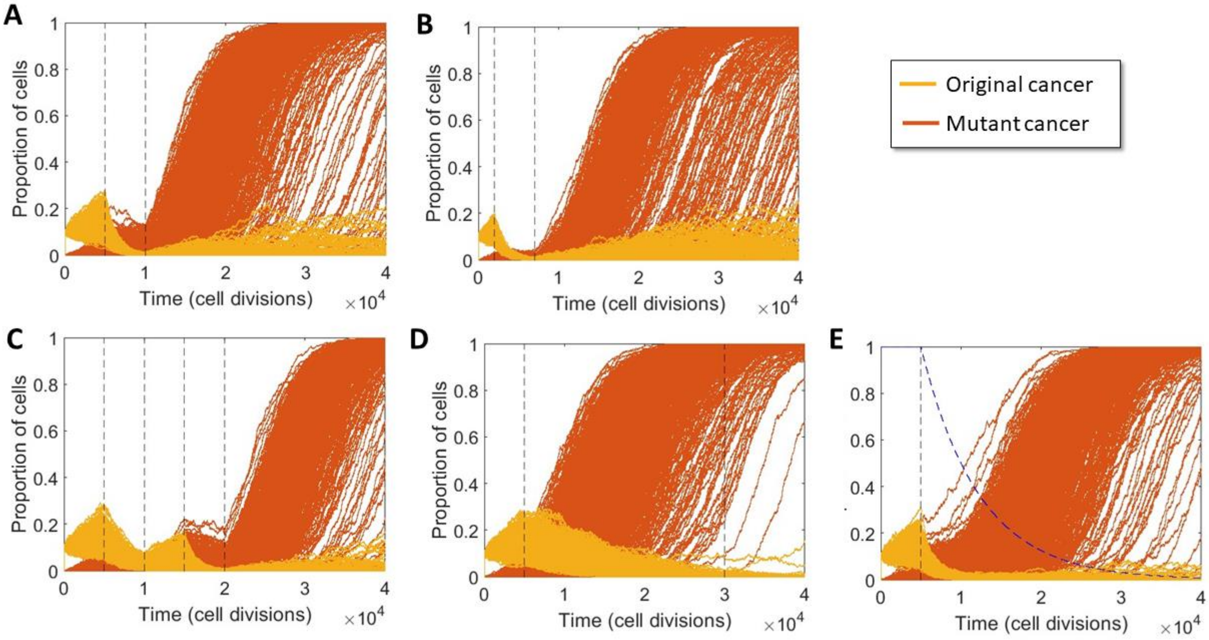

Figure S4 Supplementary figures for comparisons between simulations of different treatment methods and models. Plots of $n_{total} = 1000$ individual simulations of original and mutant cancer growths under treatment. (A, B) Single high dosages applied for 5000 cell divisions between the vertical dashed lines (A) after the mutant clone is expected to arise and (B) before the mutant clone is expected to arise. (C) Dual dosing with the first targeting the mutant cancer clone and the second targeting the original cancer clone. (D) Sustained dosing with an efficacy one fifth of that in other tests, applied over 25,000 cell divisions. (E) High dosage with a waning drug concentration modelled by the blue dashed line.

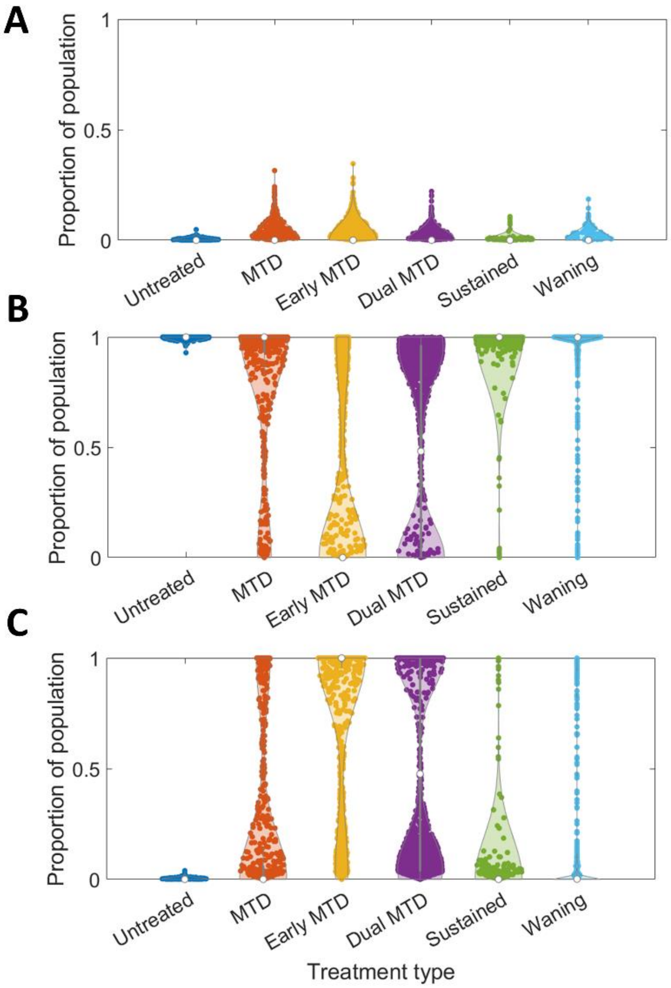

Figure S5 Supplementary figures for comparisons between final proportions of cancer under of different treatment. Final proportions of cancer growths for $n_{total} = 1000$ simulations under treatments including respectively: single. high dosages applied for 5000 cell divisions after the mutant is expected to arise and then before it is expected to arise, dual dosing with the first targeting the mutant cancer clone and the second targeting the original cancer clone, sustained dosing with an efficacy one fifth of that in other tests, applied over 25,000 cell divisions, and high dosage with a waning drug concentration modelled by the blue dashed line seen in **Figure S4E**. These treatment methods are tested simulations involving both (A) the original cancer clone, and (B) the mutant cancer clone and (C) healthy cells.

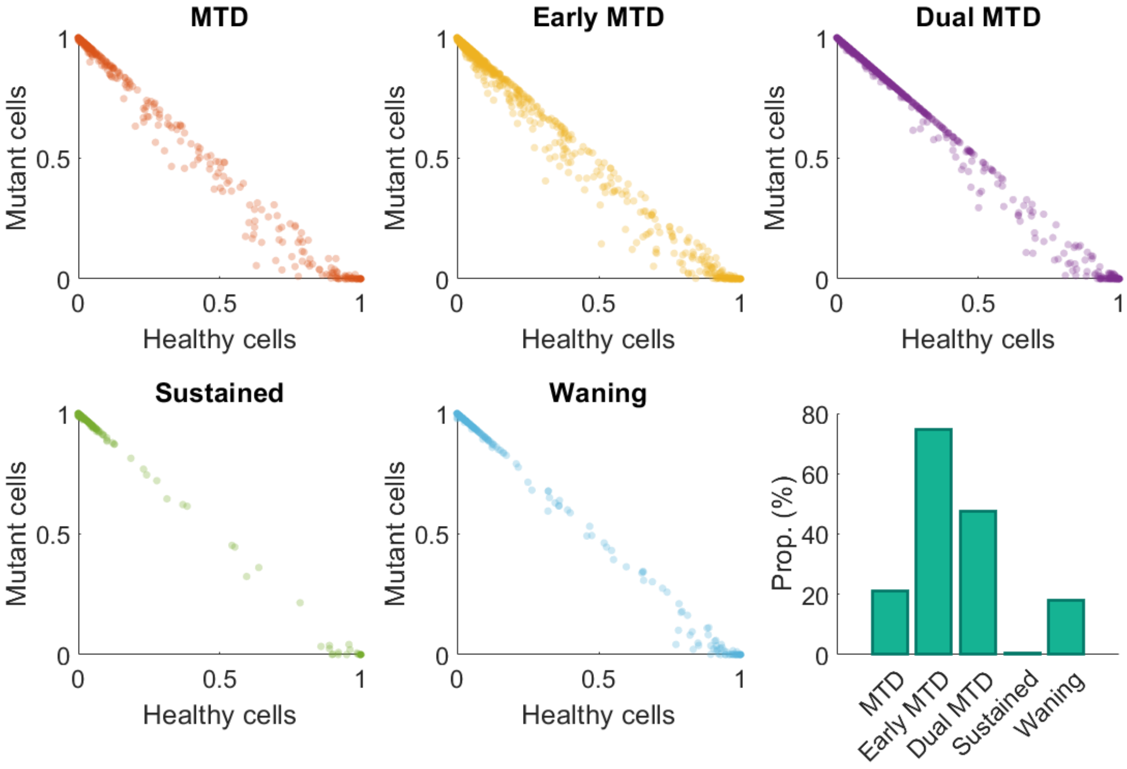

Figure S6 Supplementary figures summarising the proportion of healthy cells after varying treatment styles (corresponding to Figure S5 and Figure 5 in the main text). Final proportions of healthy cells and mutant cells for the 5 treatment styles: MTD, Early MTD, Dual MTD, Sustained and Waning, given as a scatter plot. It is clear that at the end of treatment predominantly the tumour is made up of mutant cells or healthy cells. We see survival of the original cancer cell population in MTD and Early MTD treatment scenarios, whereas other treatment styles such as the sustained treatment and wanning treatment are primarily all mutant or healthy cells. This is evident by the points all aligning on the diagonal. The final plot is a bar graph for the percentage of the simulations where the healthy cells were 100% of the cell population after treatment. From this figure, the majority of Early MTD simulations saw the healthy cells regain control of the population and recover, this can be seen in **Figure S5C** and **S4B**.

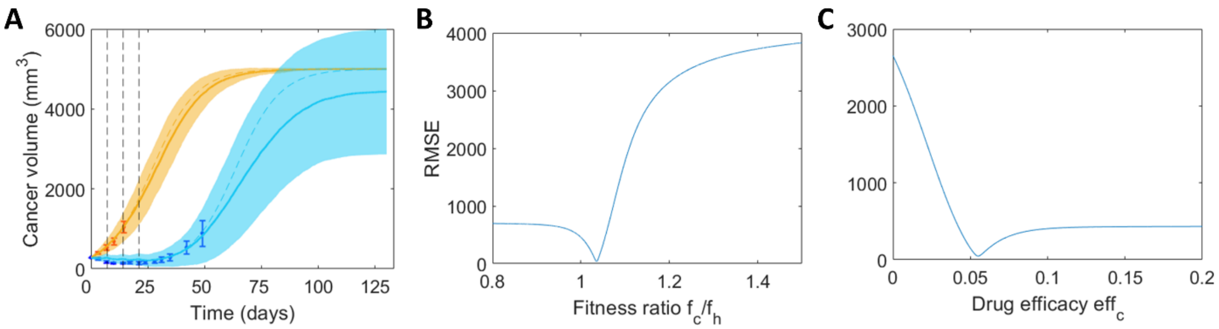

Figure S7 Supplementary figures summarising model fitting for $r_m = 0$. Fixing the mutation probability $r_m = 0$, the vehicle and treatment data [1] are fit to the recursive model in **Eq. (5)** similar to **Figure 6A** and **6B** in the main text. Comparison between data and model fits for vehicle cancer growth data (orange) and growth under treatments applied four times (blue) [12] starting at zero days and re-administered every seven days with waning drug concentration, simulations shown with mean and standard deviation over $n_{total} = 100$. The sold line and shaded region are the mean and standard deviation of the Moran model and the dashed line is the recursive model. (B) RMSE between the recursive model and the data for the fitness ratio f_c/f_h fit to the vehicle measurements. (C) RMSE between the recursive model and data for the efficacy of treatment eff_c fit to the treatment measurements using the optimal fitness ratio observed from (B). See **Table S2** for parameter values obtained.

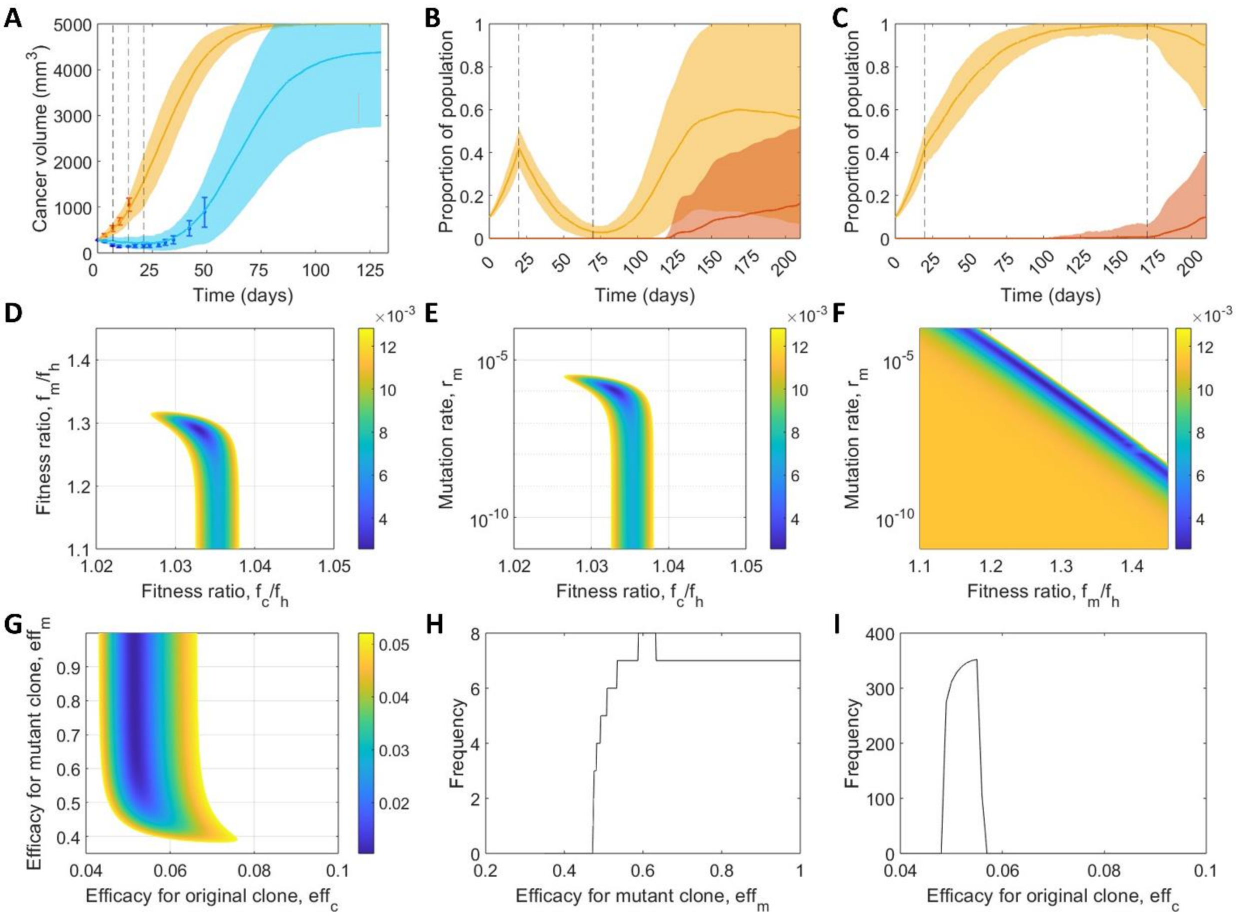

Figure S8 Supplementary figures summarising model fitting for $r_m \neq 0$. The recursive model in **Eq. (5)** was used to estimate the model parameters r_m, f_c/f_h, and f_m/f_h from the vehicle data and eff_c and eff_m from the treatment data [1]. (A) Comparison of the resulting fits for the vehicle data and treatment data presented in **Figure 6A** and **6B.** (B) The proportion of cancer cells and mutant cells in the MTD simulation in **Figure 6C**. (C) The proportion of cancer cells and mutant cells in the sustained treatment simulation in **Figure 6C**. (D-G) The RMSE as a function of two-parameter space for the (D-F) vehicle data and (G) treatment data. RMSE values plotted are those ranging from the minimum value to double the minimum value of the RMSE for that parameter space. (H-I) The frequency of eff_m and eff_c samples with the lowest RMSE.

Table S1 List of model variables and parameters. Below is a summary of all parameters/variables in the model. Relevant references for all the parameters is given.

Symbol	Description	Range
N	Total number of cells	[100,10000]
M	Total number of cell divisions	[100,100000]
f_c	Fitness of cancer cells (also applicable to all other cell types)	(1,2]
N_{c_n}	Number of cancer cells at timestep n (also applicable to all other cell types)	[0,N]
r_m	Mutation rate for type mutant clones (also applicable to other cancer mutants)	[10^{-10},0.01]
eff_c	Efficacy of treatment against cancer cells (also applicable to cancer mutants)	[0.4,0.8]
t_{start}	Starting time for treatment (in cell divisions)	[0,5000]
t_{length}	Length of treatment (in cell divisions)	[2000,5000]

Table S2 List of model parameters obtained through fitting the vehicle and treatment data from Lewis Philips *et al.* [1], see **Figure 6A** and **6B.**

**No mutation, $r_m = 0$**		**Mutation, $r_m \neq 0$**	
Parameter	Value	Parameter	Value
$f_c\ /f_h$	1.035	r_m	1.1×10^{-6}
eff_c	0.055	$f_c\ /f_h$	1.033
		f_m/f_h	1.289
		eff_c	0.052
		eff_m	0.7085

References

[1] G. D. Lewis Phillips *et al.*, "Targeting HER2-positive breast cancer with trastuzumab-DM1, an antibody--cytotoxic drug conjugate," *Cancer Res.*, vol. 68, no. 22, pp. 9280–9290, 2008.
